# Acknowledgement to Reviewers of *Pharmaceutics* in 2017

**DOI:** 10.3390/pharmaceutics10010008

**Published:** 2018-01-11

**Authors:** Pharmaceutics Editorial Office

**Affiliations:** MDPI AG, St. Alban-Anlage 66, 4052 Basel, Switzerland

Peer review is an essential part in the publication process, ensuring that *Pharmaceutics* maintains high quality standards for its published papers. In 2017, a total of 57 papers were published in the journal. Thanks to the cooperation of our reviewers, the median time to first decision was 21.4 days and the median time to publication was 47 days. The editors would like to express their sincere gratitude to the following reviewers for their time and dedication in 2017:

Adamczak, MałgorzataLiu, ZhixiaAhmad, ZeeshanLochhead, Jeffrey J.Alder, JaneLolla, DineshAl-khattawi, AliLollo, GiovannaAlmoazen, HassenLozoya-Agullo, IsabelAlzaid, FawazLu, YangAn, SaiLutton, Rebecca E.M.Anraku, MakotoMa, YongArgia, KatsuhikoMajor, IanBácskay, IldikóMalli, RolandBaki, GabriellaMandava, NandaBansal, ArvindMarchesan, SilviaBarradas, ThaísMariño, EduardoBaswan, SudhirMarkl, DanielBikiaris, DimitriosMartinez Illamola, SilviaBins, SanderMatsugaki, AiraBirch, JohnMatzke, Gary R.Bleackley, Mark R.Mazzucco, EleonoraBotre, FrancescoMiranda, PerryBrocks, Dion R.Mohammed, YousufBrudzynski, KatrinaMontenegro, LuciaBruni, GiovannaMourtas, SpyrosBurkhart, David J.Murnane, KevinBurnette, RonaldMurphy, AndrewCatanzano, OvidioMusch, MarkCavalli, RobertaNaik, ShivangiCelli, GiovanaNanavati, CharviChandasana, HardikNazzal, SamiChen, HongweiNikkhah-Moshaie, RoozbehChen, LiOtero-Espinar, FranciscoChiang, Hsiu-MeiOzawa, ShogoCho, Cheong-WeonPadrela, LuisChow, Aviva Shing FungPalavai, Sripal ReddyChu, DafengPapadoyiannis, Ioannis N.Coiffard, Laurence J. M.Patel, BrijeshkumarCollier, AbbyPatel, HardikkumarCroker, DenisePatel, NiketkumarCudic, PredragPaul, ShubhajitDaneshtalab, NorikoPawar, KasturiDansette, Patrick M.Perepelyuk, MarynaDash, RanjeetPiao, XijunDave, KaushalkumarPopat, AmiraliDavid, BerryPunzo, FrancescoDe Caro, VivianaPuranik, AmeyDearden, John C.Quodbach, JulianDeshpande, RahulRabanal, FrancescDomb, AviRae, ColinDouroumis, DennisRahib, LolaDuval, Raphael E.Rais, RanaEfthimiadou, Eleni K.Rice, JohnElShaer, AmrRitzoulis, ChristosEsteve-Romero, JosepRizzolio, F.Eu, PeterRobertson, SherrylEuaggelos, GikasRollini, ManuelaFan, WeiRosado, CatarinaFarah, ShadyRosania, GusFarrugia, BrookeSacco, PasqualeFei, Andrew Chang-YoungSau, SamareshFeng, XinSaville, DorothyFirer, MichaelSchauss, AlexanderFisher, JeffreySchmitt, Ulrich R.Gotsbacher, MichaelSehgal, InderGuada, MelissaSeidel-Morgenstern, AndreasHao, JinsongShah, KhyatiHaque, TasnuvaShah, RohanHaron, MonaShahbazi, Mohammad AliHaywood, AlisonShi, ZhenzhenHoffman, Angela M.Silva, Margarida F. B.Hofmann, UteSingh, AnilHoskins, ClareSkalko-Basnet, NatasaHyun, Bae KiSoloshonok, Vadim A.Ickowicz, AdrienSomani, SukrutIndiveri, CesareSouthworth, RichardIta, KevinSu, JingJain, GauravSubramaniam, PrasadJaiswal, JagdishSuk, Jung SooJiang, KeSun, JingjingJönsson, SivSun, WujinKakkar, AshokSun, YuanKathuria, HimanshuTakiyama, HiroshiKatsila, TheodoraTheile, DirkKatsumi, HidemasaTrandafirescu, CristinaKazunori, KadotaTseng, Ching-LiKeiser, MarkusTunaru, SorinKim, BohyungUekusa, HidehiroKim, Chun HoVan Geijlswijk, Ingeborg M.Kim, Tae KonVandooren, JenniferKleinebudde, PeterVaramini, PegahKodama, KoichiVenkataraman, ShrinivasKopel, PavelWaldmeier, FelixKorth-Bradley, JoanWang, JingKřen, VladimírWang, XinwenKubo, ToshioWang, ZhijunKucuk, IsrafilWei, ChangyongKuo, AndyWeiss, JohannaLakowski, TedWibowo, DavidLaniado-Schwartzman, MichalWinter, StefanLayek, BuddhadevWu, JianboLee, Hye SukXia, TianLee, Jae-HoXiang, DongxiLee, JaehwiXu, RaymondLeporatti, StefanoXu, Zhi Ping (Gordon)Leung, Ricky Yuet-KinYeckel, Catherine WeikartLi, MengYuba, EijiLin, ZhoumengYu-Tse, WuLitti, LucioZaghini, AnnaLiu, FeiZhang, YingyueLiu, ZhiqiuZhu, Wei

